# Friction Characteristics of Low and High Strength Steels with Galvanized and Galvannealed Zinc Coatings

**DOI:** 10.3390/ma17205031

**Published:** 2024-10-15

**Authors:** Ji-Young Kim, Seung-Chae Yoon, Byeong-Keuk Jin, Jin-Hwa Jeon, Joo-Sik Hyun, Myoung-Gyu Lee

**Affiliations:** 1 Automotive Steel Application Engineering Team, Hyundai Steel Company, 1480 Buckbusaneop-Ro, Songak-Eup, Dangjin-Si 31719, Republic of Korea; jiyoung@hyundai-steel.com (J.-Y.K.); scyoon@hyundai-steel.com (S.-C.Y.); phentas12@hyundai-steel.com (B.-K.J.); jhjeon@hyundai-steel.com (J.-H.J.); hjs401@hyundai-steel.com (J.-S.H.); 2 Department of Materials Science and Engineering & RIAM, Seoul National University, 1 Gwanak-Ro, Gwanak-Gu, Seoul 08826, Republic of Korea

**Keywords:** friction, steel sheets, zinc coating, contact pressure, sliding velocity, asperity flattening

## Abstract

As vehicle body structures become stronger and part designs more complex for lightweight, controlling frictional properties in automotive press forming has gained critical importance. Friction, a key factor in formability, is influenced by variables such as contact pressure, sliding velocity, sheet strength, and coatings. This study investigates the friction characteristics of steels with tensile strengths of 340 MPa and 980 MPa, under galvanized (GI) and galvannealed (GA) zinc coatings. Experimental results reveal that asperity flattening, a significant factor in determining friction, increases with contact pressure normalized by tensile strength, particularly for GI-coated steels. However, the relationship between friction and surface flattening deviates from conventional expectations, with the friction coefficient initially rising with increased flattening area up to ~20% before decreasing as flattening progresses. These findings suggest that traditional empirical formulas may not fully capture friction behavior under specific conditions. By understanding this inflection point, where friction reduces under high contact pressure, the study provides valuable insights for optimizing formability and improving sheet metal forming processes, especially in scenarios where precise friction control is critical for producing high-quality automotive parts.

## 1. Introduction

Due to recent advances in eco-friendly vehicle technology, materials applied to automobile parts have been diversified, and the shape of the parts has become more complex. Accordingly, advanced forming technology has been applied to manufacture automotive parts, which require the optimized formability and processing parameters in the design stage [1,2].

Various process parameters in automotive manufacturing are associated with the formability of final products. For example, the formability of steel sheets highly depends on their plastic behavior along with other press-forming parameters. The typically evaluated plastic behavior of sheet metals includes isotropic or anisotropic strength and elongation, and the key forming parameters are the blank holding force and contact between the workpiece and tools. To date, besides the experimental works on the effect of process parameters on the overall formability of the sheet parts, computational approaches such as finite element (FE) modeling and simulation have also been popular for optimization. Thus, the accuracy and numerical efficiency in the computational simulation are of critical importance [3,4,5,6,7,8].

Furthermore, considerable research has focused on understanding the role of friction in sheet metal forming, as it plays a critical role in determining the quality of the final product. Among the various processing factors, the frictional characteristics between tools and sheet metals have been extensively studied to ensure the production of high-quality sheet parts. Friction arises from complex contact conditions during forming and is influenced by factors such as sheet strength, material composition, tool materials, surface roughness, lubrication, coatings, etc. [9,10,11,12,13]. Thereby, studies reported that a conventional approach with constant friction coefficients in predicting the formability of automotive steel sheets could not give accurate results, while more advanced friction models, such as those based on variable friction coefficients, should be further developed [1,14,15,16]. For example, friction modeling with variable coefficients as a function of contact conditions during press forming increased the accuracy of formability prediction [15,16,17].

As the main factors influencing the friction in the sheet metal forming process, the contact pressure and sliding velocity are related to the blank holding force and forming speed [18,19,20,21,22]. For example, the study on the effect of forming speed on the friction coefficient of 60 K grade steel plate showed that the friction coefficient decreased as the sliding speed increased under low contact pressure. Also, friction tests for various sheet metals at a high drawing speed of 600 m/min or faster also reported that the friction coefficient decreased as the drawing speed increased. Other studies reported that the lubricating oxide can be formed on the material surface if the surface temperature increases during press forming. This resulted in reduced shear strength between tools and sheet metals. As for the effect of contact pressure on the friction coefficient, cold-rolled high-strength steel exhibited a decreased friction coefficient as the contact pressure increased at the region of low contact pressure [10,21]. However, other studies also reported that the effect of contact pressure varied and was strongly dependent on the material properties and contact conditions [10,23,24,25,26]. Particularly, friction depends on the material’s hardness, surface roughness, or evolution during formation.

Bowden and Tabor [27,28] studied the effect of contact asperity on frictional behavior, and they observed that a linear relationship between contact pressure and friction coefficient changes to non-linear as the real contact area becomes large. They also found that the bulk plastic deformation changed the formation of the contact area and, eventually, the friction coefficient. Orowan et al. [29] investigated the relationship between surface contact area and stick–slip behavior, focusing on the normal load and friction coefficient.

Moreover, the type and characteristics of coatings applied to sheet metals significantly influence friction and formability [30]. Zinc coatings demonstrate this impact in two main aspects. First, depending on the coating type, surface roughness varies, affecting the contact area. Second, changes within the coating layer alter the shear yield strength of zinc-coated layers. For instance, the tensile yield strength of the coating increases with thicker Fe-Zn intermetallic phases, leading to higher shear yield strength and, consequently, an increase in the friction coefficient [31]. The formability of zinc-coated sheet metals is influenced by a complex interplay of surface friction, coating adhesion, and strain state distribution [32,33,34]. Surface friction affects deformation modes and can compromise sheet metal formability. Additionally, the brittleness of the coating layer leads to varying adhesion under different contact conditions. Therefore, to accurately predict the formability of galvanized steel sheets, it is essential to first understand the coefficient of friction and friction behavior based on contact conditions and the physical properties of the coating layer.

Based on the previous studies on the friction characteristics in terms of the material properties’ contact conditions, the present work aims to further investigate the friction behavior of steel sheets with different strength grades, types of coating, and contact conditions. Thus, steels with significantly different tensile strengths and two zinc-coated surfaces are considered in this study. The frictional behavior is particularly related to the contact surface change between the tool and sheet metal during the press forming process. For this purpose, three sliding speeds and three normal loads are applied to the two steel sheets. These distinctive test conditions enabled us to realize various contact conditions and surface flattening of steel surfaces, which affect the characteristics of frictional behavior. Finally, experimental results are analyzed based on the microscopic pattern of asperity flattening and compared to the classical adhesion theory.

## 2. Materials and Methods

### 2.1. Materials and Sample Preparation

**Figure 1:**
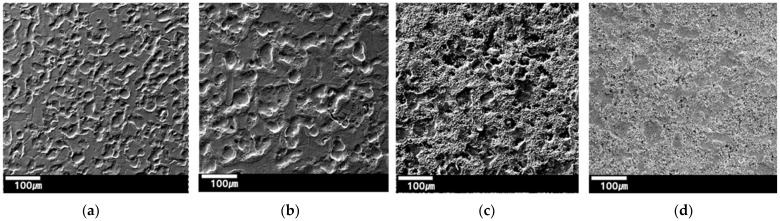
The top view of the four as-received specimen surfaces: (**a**) TS340-GI, (**b**) TS980-GI, (**c**) TS340-GA, and (**d**) TS980-GA.

Two steel sheets with different tensile strengths, 340 MPa and 980 MPa were investigated. Their coating conditions were either galvannealed (GA) or galvanized (GI). Therefore, four types of specimens were prepared in total and were labeled as TS340-GI, TS980-GI, TS340-GA, and TS980-GA, indicating both tensile strength and coating type for each specimen. The thickness of the TS340-GI and TS340-GA specimens was 0.7 mm, while the thickness of the TS980-GI and TS980-GA specimens was 1.0 mm. TS340 steel sheet is commonly used for automotive outer panels, and TS980 steel sheet is a representative grade primarily applied to automotive components requiring high stiffness. The GI coating involves applying a layer of zinc to steel sheets. GA is a type of coating applied to steel sheets through a process that involves galvanizing followed by annealing. GI and GA coatings are both commonly used in various industries, including automotive, construction, and appliance manufacturing, where corrosion resistance is crucial. Figure 1 shows the surface morphology of the four as-received specimens, and they clearly represent different initial surface conditions depending on both strength and coating. Table 1 shows the sample details. The strengths of base materials, as well as the strengths and hardness of the coatings, are listed in Table 1. The hardness of the coatings was measured using a Vickers hardness tester (Mitutoyo HV-100, Mitutoyo, Kawasaki, Japan) following ISO 6502-2 standards [35], and for comparison with substrate strength, the hardness was converted into tensile strength [36,37].

TS340-GI and TS980-GI have similar surface topography, with a lower average roughness (Ra) and maximum peak-to-valley height (Rz) compared to TS340-GA and TS980-GA, as shown in Table 1. The skewness (Rsk) values also reveal that the GI coatings exhibit more pronounced valleys on the surface, which could enhance lubricant retention. In contrast, the GA coatings show different characteristics, with TS340-GA having a moderately negative skew and TS980-GA showing a balanced surface topography, indicating an even distribution of peaks and valleys.

**Figure 2:**
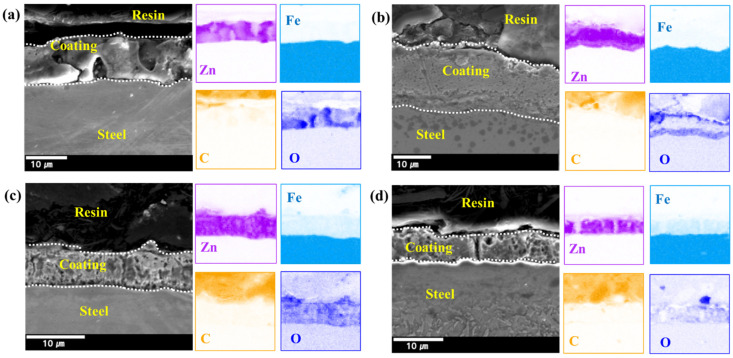
The cross-sectional view and EDS images of the four as-received specimen surfaces: (**a**) TS340-GI, (**b**) TS980-GI, (**c**) TS340-GA, and (**d**) TS980-GA.

The original cross-sectional view of GI and GA coatings is presented in Figure 2. As shown in Figure 2, the GI coating consists solely of zinc, whereas the GA coating is formed as a zinc-iron alloy. Note that the strength and hardness of GA coating is much higher than that of the GI coating. The GI and GA coatings’ thickness was approximately 13 μm and 6 μm, respectively. The rectangular-shaped specimens for the friction test were cut from the sample in the transverse direction to the roll. Steel sheets produced through a continuous rolling process typically exhibit isotropic roughness. However, slight variations in roughness and mechanical properties have been observed depending on the direction of rolling. In our study, it was recognized that the transverse direction (TD) to rolling had a higher coefficient of friction than RD. Therefore, despite these minor variations in roughness and friction coefficient across different directions, standard steel sheet product measurements are conducted in the transverse direction. This approach ensures a conservative estimate of the friction behavior in the industry. For the tests, the sample size was 220 mm × 30 mm (length × width). Before each friction test, the specimen was pre-treated following the standards ISO27831-1 [38] and ISO22462 [39]. First, any contaminants on the specimen surface were removed with ethanol solvent. Then, two drops of lubricant were applied to the specimen surface and then wiped with a clean cloth. The lubricant used in the present work was BW-80HG, and the lubrication amount applied was 2000 mg/m^2^. The kinematic viscosity of the lubricant is 14.0 mm^2^/s at 40 °C, and the base oil of the lubricant is composed of hydrotreated light paraffinic, hydrotreated heavy paraffinic, and sulfonic acids–petroleum–barium salt. We used the kinematic viscosity at 40 °C because, in continuous press processing, the die or punch temperature rises as the number of pressings increases.

### 2.2. Friction Test

**Figure 3:**
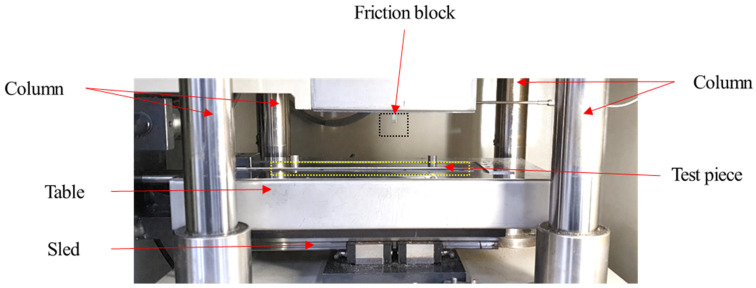
A single-sided sliding type friction test equipment.

The sliding friction test with a single-side contact was conducted. The test equipment, which is a single-sided sliding type of friction equipment, is shown in Figure 3. The test equipment is shown in Figure 3. All tests were carried out at 23 °C and 50% relative humidity (RH) according to the guideline of ISO standard [39]. Constant loads of 300, 1000, 3920, and 8000 N were applied during the friction tests. These correspond to the contact pressures of 10, 33, 130, and 267 MPa, considering the contact area of 30 mm^2^. Before each test, the uniformity of contact pressure on the test specimen was checked with photosensitive paper. The reason for conducting coefficient of friction measurements across a range of contact pressures, from 10 MPa to over 100 MPa, in this study is to encompass the various forming conditions that can occur during the forming processes of automotive components. In actual press forming of automotive parts, most areas experience relatively low contact pressures in the range of a few MPa. However, high contact pressures may arise in certain zones of the stamping die, particularly at sharp corners with small radii or localized regions undergoing significant deformation [40]. Therefore, the range of contact pressures was selected to reflect these critical areas. Similarly, the sliding velocity conditions were determined based on forming analyses of several representative automotive outer panel components. The relative sliding velocity between the tool and specimen was set to be 0.05, 0.2, and 0.9 m/min. A constant sliding distance of 100 mm was applied to all tests. The tool material used in the present work was X153CrMoV12 steel and was ion nitrided after vacuum heat treatment with a nitride layer of about 0.07 mm. The roughness and hardness of the friction tool block were 0.1 Ra and 1000 Hv, respectively. During the friction test, the tool block was not re-polished due to the considerable difference in hardness between the tool and the material. The tool, made of SKD 11, was vacuum heat-treated and ion-nitrided, achieving a surface hardness of approximately 1000 Hv, which is substantially higher than the hardness of the Zinc coated specimens. This substantial hardness difference minimizes potential changes in the tool surface roughness over the course of the tests. The tests were conducted using the tool without re-polishing, and specifically, we conducted three repetitions for each of the nine test conditions on the same material, resulting in a total of 27 tests performed without re-polishing the tool. The applied load and contact pressure were normalized by the contact area and tensile strength (TS), respectively, using Formula (1) to exclude the material strength’s effect, which is summarized in Table 2.

(1) $$Normalised\;Contact\;Pressure\left(NCP\right)=\frac{Applied\;load(N)}{Contact\;area\left({mm}^{2}\right)\times Tensile\;strength\left(M|P|a\right)},\;$$

As a result, a proportionally higher load was applied for materials with higher tensile strength, such as TS980 steel, compared to lower-strength materials like TS340 steel. This approach allows for a more accurate comparison of frictional behavior across different material grades. However, due to equipment limitations, where the applied load exceeded the operational range, a lower contact pressure was applied to the TS980 steel than to the TS340 steel under the 130 MPa condition

The friction coefficient (μ) was calculated from the normal, and friction forces were recorded during sliding. At least three tests were repeated for each test condition. The coefficient of friction is determined using the following Equation (2):

(2) $$\mu =\frac{F_{f}}{F_{n}},$$

where $F_{f}$ is the friction force, and $F_{n}$ is the normal force.

### 2.3. Sliding Surface Analysis

After each friction test, the sliding surface of the steel sheet was observed using a scanning electron microscopy (SEM) with a Phenom Desktop SEM from Thermo Fisher Scientific (Waltham, MA, USA) and three-dimensional (3D) confocal imaging equipment using ADE’s MicroXAM. The SEM specimens were cut in the direction parallel to the sliding surface, and the area of flattened asperities was evaluated by measuring the height distribution using the 3D confocal image analyzer.

The SEM analyzed the sliding surface and cross-section of the tested specimen, including the surface deformation and flattening of asperity, with varying sliding velocity and normal force. Additionally, the cross-section was analyzed for elemental composition using energy-dispersive spectroscopy (EDS). The asperity height corresponding to the local maxima is defined as the transition height, and the flattening area αh is determined as follows [41].

(3) $$\alpha_{h}=\int_{h_{t}}^{\infty }\varphi_{d}\left(z\right)dz,$$

where *h*_*t*_ and *φ_d_* (%) are the height of the asperity corresponding to the local maximum peak and normalized height distribution of the deformed surface, respectively. A median filter with a kernel size of 3 × 3 was used for the measurement area of 2 mm × 2 mm.

## 3. Results

### 3.1. Effect of Strength and Coating Condition on Friction Coefficient

**Figure 4:**
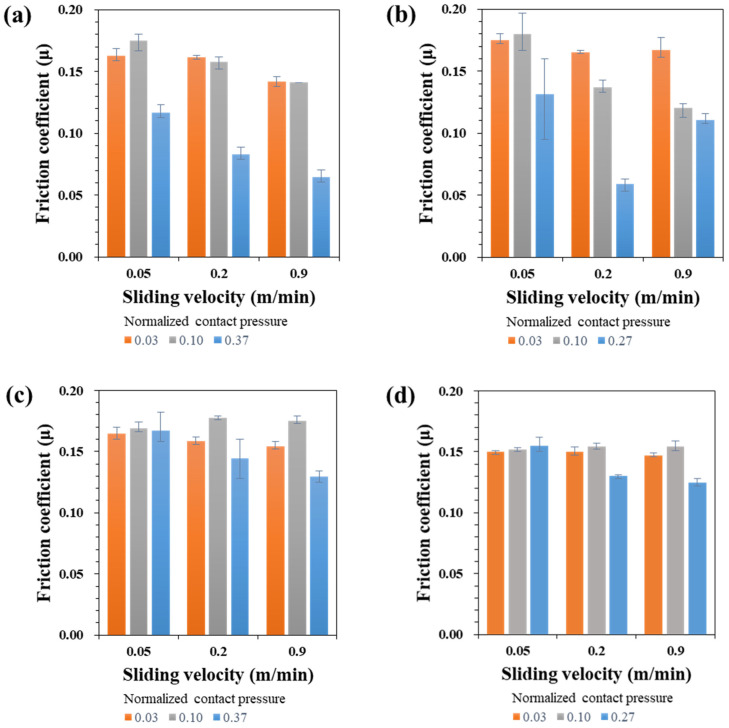
Measured friction coefficients with respect to the sliding velocity and normalized contact pressure: (**a**) TS 340-GI, (**b**) TS 980-GI, (**c**) TS340-GA, and (**d**) TS980-GA.

The measured friction coefficients of the four types of specimens are summarized in Figure 4 under various sliding velocities and normalized contact pressures.

For the TS340-GI specimen, the friction coefficient decreases as the sliding velocity increases. As shown in Figure 4a, under the normalized contact pressure (NCP) of 0.03, the friction coefficients decreased from 0.163 to 0.142 as the sliding velocity was increased from 0.05 to 0.9 m/min. Regarding the effect of the contact pressure, the change of friction coefficient was uni-modal. When the normalized pressure increased from 0.03 to 0.1, the change in friction coefficient was marginal. However, a significantly reduced friction coefficient was observed when the normalized contract pressure increased as high as 0.37. For example, at the sliding velocity of 0.05, the coefficient of friction changed around 10% when the normalized contact pressure changed from 0.03 to 0.1, while it decreased over 30% for the normal contact pressure of 0.37. For the steel with higher strength and the same GI coating condition as TS980-GI, Figure 4b shows generally similar results in terms of the effect of the sliding velocity and contact pressure on friction coefficient as those of TS340-GI. The difference is that the friction coefficient is less sensitive to the sliding velocity, especially when the normalized contact pressure is as low as 0.03.

The friction coefficients of GA-coated steel sheets were less sensitive to the sliding velocity and normalized contact pressure than those of GI-coated steels, as shown in Figure 4c,d. This is particularly clear when the normalized contact pressure was an intermediate value of 0.1. That is, for the GI-coated steels, the friction coefficient exhibits an obvious decrease if the sliding velocity increases, while the friction coefficients of GA-coated steels under the contact pressure of 0.1 are virtually constant regardless of material strength. The commonly observed decrease of friction coefficient with increased sliding velocity was observed in GA-coated steels only when the normalized contact pressure was as high as 0.37 and 0.27 for TS340 and TS980 steel, respectively. Another noticeable result is that the friction coefficient variation in processing factors (sliding velocity and contact pressure) is much smaller for the GA-coated steels than that of the GI-coated steels. The GI-coated steels present friction coefficients in the range of 0.059 to 0.18, but the range becomes 0.125 to 0.177 for GA-coated steels. Moreover, the variation in the level of friction coefficient does not significantly depend on the strength of the steel.

### 3.2. Characteristics of Frictional Behavior

**Figure 5:**
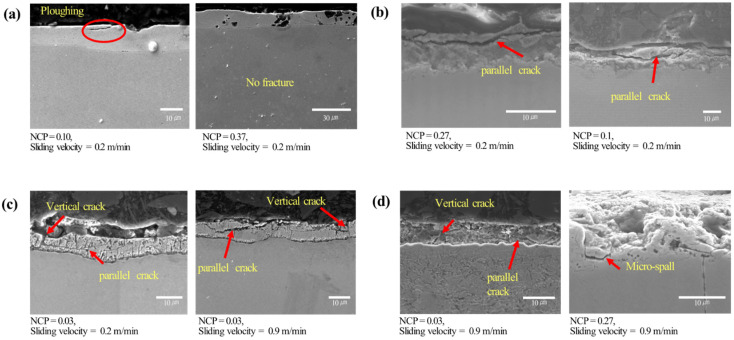
SEM measured cross-sectional images of the test specimens: (**a**) TS340-GI, (**b**) TS980-GI, (**c**) TS340-GA, and (**d**) TS980-GA. NCP denotes the normalized contact pressure.

**Figure A1:**
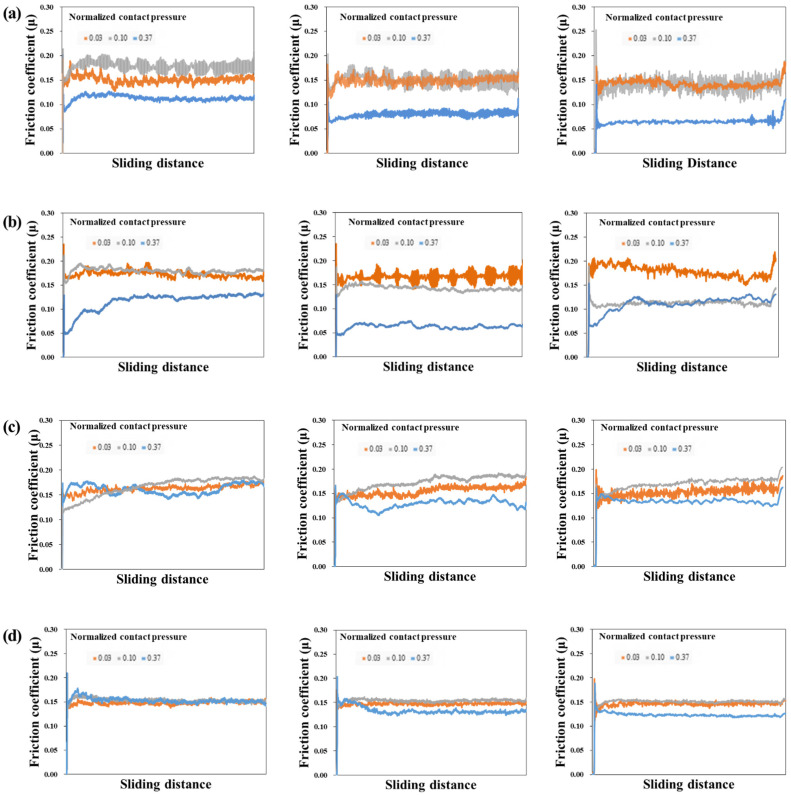
Comparison of Friction graphs with respect to sliding velocity and contact pressure: (**a**) TS340-GI, (**b**) TS980-GI, (**c**) TS340-GA, and (**d**) TS980-GA. For each coating condition, three sliding velocities are considered as (**left**) 0.05 m/min, (**center**) 0.2 m/min, and (**right**) 0.9 m/min.

More detailed analyses of the characteristics of frictional behavior were performed for the tested specimens. Besides the friction coefficient, the friction modes and the existence of wear and surface fracture were quantified. Also, they were correlated to the sliding velocity, normalized contact pressure, material strength, and coating types. The characteristics of these frictional behavior are summarized in Table A1a–d in Appendix A. The symbols X, △, and O represent “barely observed”, “occasionally observed”, and “almost always observed”, respectively. “Barely observed” was applied when 1 or fewer instances were found, “occasionally observed” when 2 to less than half of the instances were found, and “almost always observed” when more than half of the instances were found. As supplementary data, the raw graphs of friction coefficient change during sliding are also given in Figure A1a–d in Appendix A, which clarifies the occurrence of stick–slip behavior during the friction test. In addition, the microscopic analyses were presented using SEM, which observed the deformation and fracture of coating layers, as shown in Figure 5a–d.

The deformed surfaces of TS340-GI steel under different contact pressures are shown in Figure 5a, where flattening and plowing (or scratching) of the coating layer can be observed. As presented in Table A1a, the increased contact pressure resulted in larger asperity flattening and deformation of the coating layer. On the other hand, the effect of sliding velocity on asperity deformation was more complicated. For example, at the normalized contact pressures of 0.1 and 0.37, the increased sliding velocity led to reduced asperity flattening. The stick–slip phenomena were observed only at the normalized contact pressure of 0.03 and 0.1. The existence of stick–slip can be referred to as the serrated friction curve shown in Figure A1a. The high friction coefficient of TS340-GI under this contact pressure can be attributed to this stick–slip behavior. The stick–slip was hardly observed at higher normalized contact pressure. Besides, no crack was observed in the coating layer of TS340-GI for all tested conditions.

The friction characteristics of TS980-GI steel in terms of asperity flattening and stick–slip behavior were similar to those of TS340-GI steel (see Figure 5b and Figure A1b). However, unlike the TS340-GI steel, evident parallel cracks in the coating layer were noticeable in TS980-GI steel in all experimental conditions (Figure 5b). The parallel cracks in TS980-GI steel during sliding may change the state of material-tool contact, which ultimately results in the reduction of stick–slip behavior compared to TS340-GI steel.

The frictional behavior of TS340-GA steel presented less asperity flattening and stick–slip than those of TS340-GI steel, as shown in Figure A1c.

From the previous references [24,25], it is known that the stick–slip behavior encountered during sliding contact can be caused by either adhesion or asperity interlocking. Enhanced chemical compatibility, increased contact area, and prolonged residence time can increase the adhesion. On the other hand, asperity interlocking occurs when a rigid asperity penetrates a softer asperity, which can be alleviated by either elevating the hardness of the softer material or reducing the applied load. The reduced stick–slipped TS340-GA-coated steel might be due to the increased hardness of the coating and decreased adhesion caused by the asperity interlocking. Also, less flattening can be related to decreased adhesion. In contrast to the GI-coated steels, the coating surface of TS340-GA steel exhibited obvious vertical cracks (Figure 5c and Table A1c). This is closely related to the strength of the coating layer because the vertical cracks are predominantly caused by tensile stress along the direction of frictional sliding [42]. Like the GI-coated steels, the GA-coated steels presented similar frictional characteristics, as shown in Table A1c. However, the GA coating on higher-strength steel, like the case of TS980-GA, showed increased vertical and parallel cracks with increased loading and sliding velocity. In addition, it was found that the coating layer’s delamination (or spalling) occurred when parallel and vertical cracks occurred simultaneously, as shown in Figure 5d [43,44]. It appears that the cracks are first initiated on the surface of the coating layer and subsequently propagate in parallel, ultimately leading to the detachment of the coating layer.

Finally, it is validated that the deformation of the coating layer is intensified as the normal load increases, as explained by the evidence of plowing. While it has been reported that substrate deformation can occur as a consequence of sliding when the surface is coated [45], our present study indicates minimal signs of substrate deformation (Figure 5). Consequently, it is inferred that the increase in friction caused by substrate deformation is expected to be insignificant.

### 3.3. Analysis of Asperity Flattening on Sliding Surface

It has often been reported that the flattening of surface asperity is one of the major factors in determining contact characteristics and frictional force during sliding [23,24,25]. According to previous studies, the friction coefficient decreases as the contact normal force increases [18]. In this section, the friction behavior is further analyzed in relation to the asperity flattening for different zinc-coated steel sheets.

**Figure 6:**
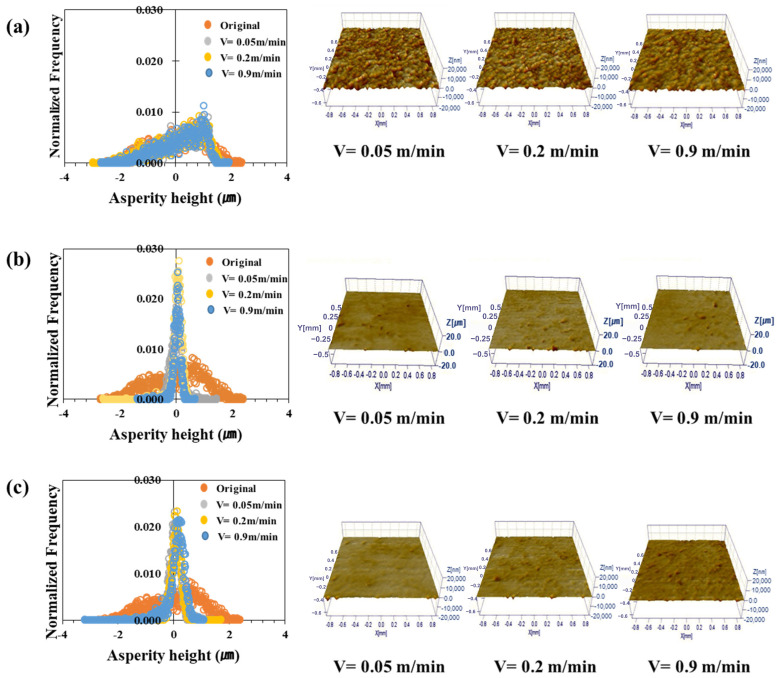
Asperity height distribution and 3D surface roughness images after friction tests on TS340-GI under different normalized contact pressures: (**a**) 0.03, (**b**) 0.1, and (**c**) 0.37.

**Figure 7:**
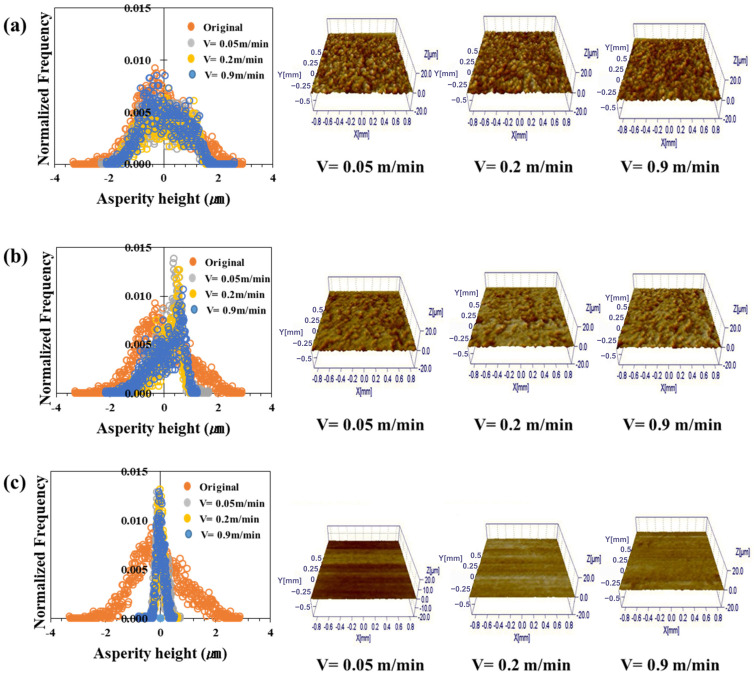
Asperity height distribution and 3D surface roughness images after friction tests on TS340-GA under different normalized contact pressures: (**a**) 0.03, (**b**) 0.1, and (**c**) 0.37.

**Figure 8:**
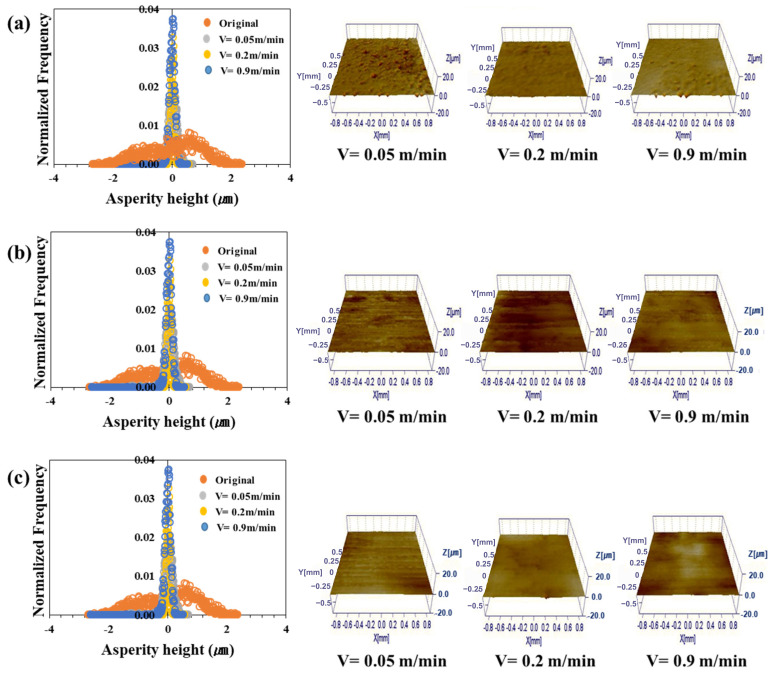
Asperity height distribution and 3D surface roughness images after friction tests on TS980-GI under different normalized contact pressures: (**a**) 0.03, (**b**) 0.1, and (**c**) 0.27.

**Figure 9:**
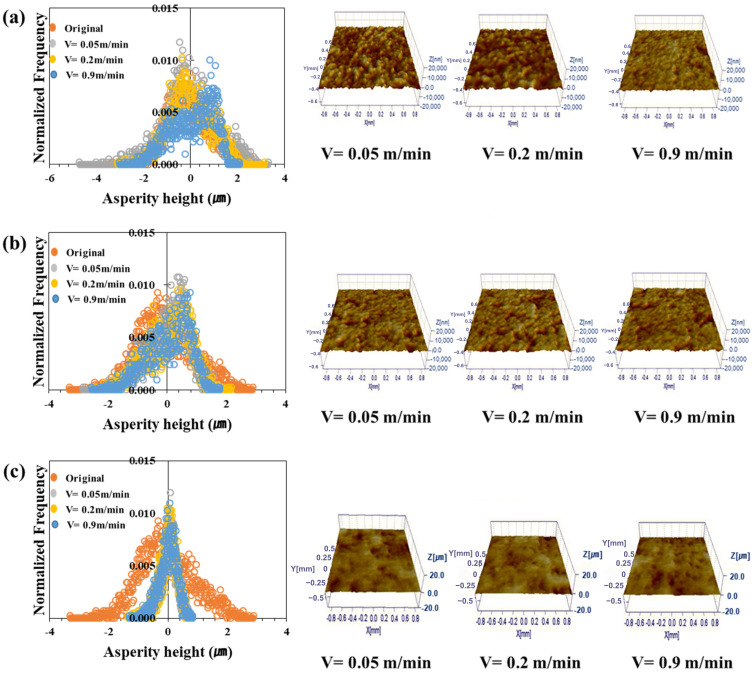
Asperity height distribution and 3D surface roughness images after friction tests on TS980-GA under different normalized contact pressures: (**a**) 0.03, (**b**) 0.1, and (**c**) 0.27.

Figure 6, Figure 7, Figure 8 and Figure 9 show the roughness images of flattened surfaces and asperity height distribution measured by the 3D confocal microscopy. The experimental results suggest that differences in surface roughness deformation appear to be influenced by the coating and substrate strength. However, since the substrate strength normalized the contact pressure, the increased flattening of surface roughness in materials with higher substrate strength may simply be a result of this normalization. Therefore, it is difficult to conclude that the substrate strength itself directly affected the roughness deformation.

Figure 6 presents the 3D confocal microscopy results for the TS340-GI specimen. Each height distribution was averaged from the data obtained at four different locations. Also, the different colors in the figure indicate the surface roughness for the sliding velocities of 0.05 m/min, 0.2 m/min, and 0.9 m/min. The surface roughness of the as-received specimen before the friction test was also included in the figure for comparison purposes. Figure 6a–c represents the surface roughness of three different contact pressures. The figures show that the change in asperity distribution noticeably depended on the contact pressure. On the other hand, sliding velocities appear to have less impact on the asperity distribution except under the high contact pressure. It was confirmed that the asperity deformation decreased as the sliding speed increased under high contact pressure. For example, the distributions of surface asperity were virtually similar for all sliding velocities when the normalized contact pressure was 0.03 (Figure 6a), but the surface roughness became much flattened with a very narrow distribution when the normalized contract pressure increased over 0.1 (Figure 6b,c). The asperity height distributions of TS340-GA for various contact conditions are depicted in Figure 7. A similar trend was noticed in the case of the TS340-GA specimen. In contrast to the findings from the TS 340-GI in Figure 6, the flattened surfaces on TS340-GA were less pronounced.

For the TS980 steel, the flattening of asperity was much more significant for GI-coated specimens (Figure 8) than for the GA-coated specimens. The GI-coated surface formed on higher-strength steel showed considerable surface deformation even under the normalized contact pressure of 0.03. However, a much higher normalized contact pressure of 0.27 was necessary to flatten the asperity for the GA-coated specimens.

## 4. Discussion

The primary aim of this study is to examine the effect of sliding contact conditions on the friction behavior of steels with different strengths and coatings and to identify the factors that contribute to the change of friction coefficient. For these purposes, in Section 4.1, we first analyzed the relationship between the coating type and friction behavior of the two steels with different tensile strengths. Here, we tried to confirm the validity of the conventional adhesion theory with an analysis of the stick–slip behavior at various contact pressures and sliding velocities. In Section 4.2 and Section 4.3, the friction characteristics were discussed based on the observed asperity flattening as a result of contacts with different sliding conditions, which varied the friction coefficient.

### 4.1. Effect of Coating and Base Metal Properties on Friction Behavior

In terms of the effect of coating on friction observed in Figure 5, cracks propagated through the substrate surface of GA-coated steels regardless of the strength of the steel. Therefore, this may indicate that the strength of the coating layer has a larger effect on the formation of cracks than the other factors [46,47]. Tensile traction applied horizontally during sliding contact has been widely recognized as one of the primary factors for crack initiation and propagation. Under heavily loaded conditions, friction induces shear and plowing, which result in tensile stress within the coating layer, while compressive stress ahead of the sliding tool. The severity of this process escalates with an increase in both the hardness of the coating layer and the applied vertical load. If the coating layer becomes more brittle with higher hardness, it is more prone to fracture under tensile stress through coating thickness [48].

**Figure 10:**
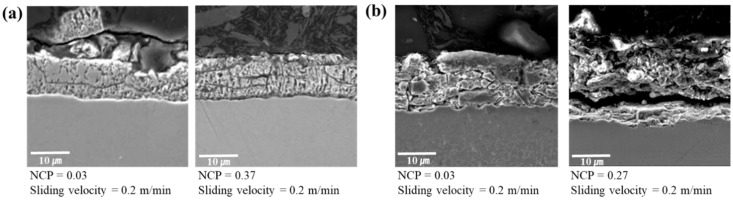
Comparison of coating layer cracks from friction tests: (**a**) TS340-GA, and (**b**) TS980-GA. NCP denotes the normalized contact pressure.

Meanwhile, the parallel cracks are known to be caused by stress accumulated through the sliding contact load in the coating layer. The stress can be quantified with the equivalent stress (typically defined as the von Misses stress), which increases as the applied load and sliding speed increases. The present study also validated it by showing increased parallel cracks as the contact pressure and sliding velocity increased. This is because parallel cracks are greatly affected by the shear and tensile stress simultaneously [49,50]. In our study, it was also confirmed that a larger number of fragmented cracks occurred in GA-coated steels than in GI-coated steels. This can be attributable to the higher hardness of GA coating than the GI coating, which leads to increased equivalent stress under contact surfaces [51]. Additionally, spallation by the delaminated coating layer initiated from parallel and vertical cracks was observed simultaneously [43]. Furthermore, more cracks were observed in the TS980 material (as shown in Figure 10). This is likely because the higher strength of the base metals reduces deformation under vertical loading, leading to increased stress concentration in the coating layer. Therefore, as the substrate strength increases, coating layer delamination and debris formation also appear more likely to increase.

Adhesion can be an influencing factor for wear under sliding contact [28,52]. This study also showed adhesion behavior in the form of stick–slip response in the measured friction coefficient (See Figure A1 in Appendix A). The stick–slip is a phenomenon that occurs as evidence of adhesion. In the stick–slip motion, when force is applied to initiate sliding between the two surfaces, they initially stick together due to static friction. As the force continues to build, the static friction is overcome, and the surface suddenly slips or moves relative to each other. Compared to the GA-coated steels, the stick–slip behavior was predominantly observed in GI-coated steels. Meanwhile, the GI-coated steels showed a noticeable decrease in the friction coefficient in the region where adhesion (or stick–slip) was reduced.

As shown in Figure A1a, the TS340-GI specimen exhibited an increased adhesion (or stick–slip) as the normalized contact pressure increased from 0.03 to 0.1 for all three investigated sliding velocities. However, when the normalized contact pressure was increased to 0.37, the stick–slip decreased rapidly despite the flattening area increased. Also, in the case of the TS980-GI specimen, the stick–slip was most pronounced when the normalized contact pressure was 0.03, but it was reduced as it was increased to 0.27. This behavior was similar to the case of TS340-GI steel. These results are inconsistent with the commonly reported adhesion theory, because the real contact area and friction force increase simultaneously when the contact pressure increases, according to Orowan et al. [26,28]. However, some previous studies have suggested that under high load conditions, the deformation of asperities due to the high load can reduce the potential energy barrier for adhesion. In other words, as the vertical load lowers the roughness, the energy barrier decreases, which can lead to reduced friction [53,54].

In terms of the sliding velocity, Boden and Leben [55] reported that the relationship between adhesion and contact state (in terms of contact time and area) was proportional. Hence, the adhesion increased when the sliding speed was reduced with the increased contact time. In this study, as consistent with previous research, TS340-GI steel showed greater stick–slips at 0.05 m/min and 0.2 m/min conditions under 0.1 normalized contact pressure. However, the largest stick–slip was measured in TS980-GI steel at the sliding velocity of 0.2 m/min and normalized contact pressure of 0.03. Therefore, it seems that more complex effects, such as surface roughness and hardness, should also be considered when analyzing the stick–slip behavior [56]. In several studies, the friction coefficient decreased as the contact pressure and sliding velocity increased [9,18]. However, other studies also reported that the friction coefficient was highly dependent on the specific contact condition and material properties. For example, some researchers measured reduced friction with increasing contact pressure and lower surface roughness under lubrication. This is known to occur under the micro-EHL condition, where peaks of the most prominent asperities are removed, causing improved local hydrodynamic load-carrying capacity. The micro-EHL effect is characterized as thin-film lubrication since the height of the surface roughness is comparable to the film thickness. In the micro-EHL model, the friction coefficient tends to decrease as the contact area decreases under boundary lubrication conditions [57,58,59,60,61].

### 4.2. Summary of Sliding Velocity and Contact Pressure on the Asperity Flattening

**Figure 11:**
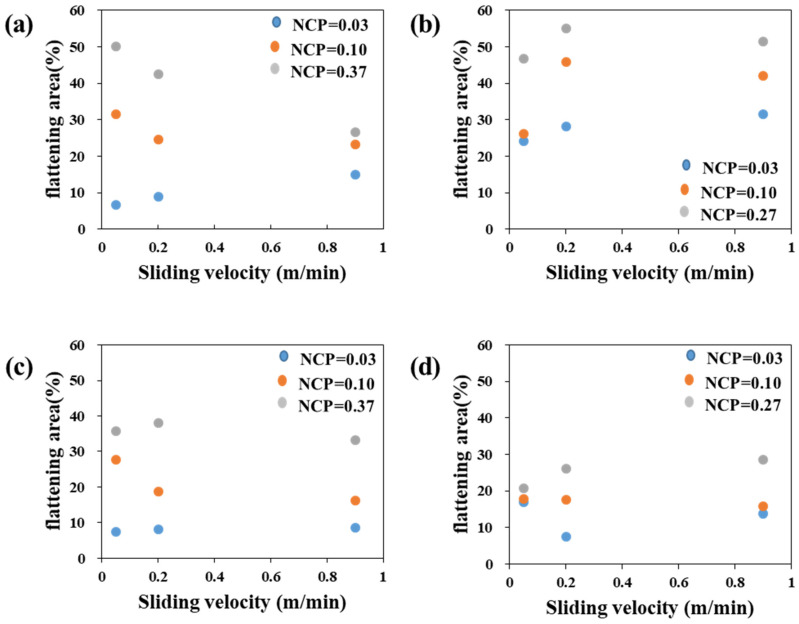
Measured flattening area (%) in terms of the sliding velocity and normalized contact pressure: (**a**) TS340-GI, (**b**) TS980-GI, (**c**) TS340-GA, and (**d**) TS980-GA.

The asperity flattening is one of the root causes of increasing friction between material and tools. To explain the change of the asperity flattening in the two investigated steel sheets, the percentage of the flattened area is plotted in Figure 11 for various contact conditions. In the figure, as the contact pressure increases, the flattening area increases. For the TS340-GI case, as the sliding velocity increased, the flattening area decreased under the normalized contact pressure over 0.1. This result appears to be related to the local side flow of the lubricant around asperities, resulting in the reduction of the lubricant film thickness with the increased sliding velocity [62]. The relationship between the sliding velocity and flattening area is still unclear in this study. The current study also showed that the increase in asperity flattening with increased contact pressure was more obvious in the GI steels. In the case of the GA-coated steels, the asperity flattening was less than that of the GI-coated steels under similar contact pressure. This is because of the higher strength of GA coating than the GI coating. The analysis can also be associated with a larger friction coefficient variation in the GI steels than that of the GA steels, regardless of the strength of the substrates (Figure 4).

### 4.3. Summary of Asperity Flattening on Friction Coefficient

Contrary to the classical analysis, the friction coefficient was not always linearly proportional to the asperity flattening. Figure 6, Figure 7, Figure 8 and Figure 9 revealed a significant decrease in roughness with increased load. Moreover, there was a tendency for the friction coefficient to decrease as the load increased. Thus, as observed in a previous study, in a lubricated state, the flow can generate supporting force against the load, and under a larger load, it reduces direct contact between the asperities, thereby reducing the effect of asperity contact on friction and leading to lower friction coefficients [63].

**Figure 12:**
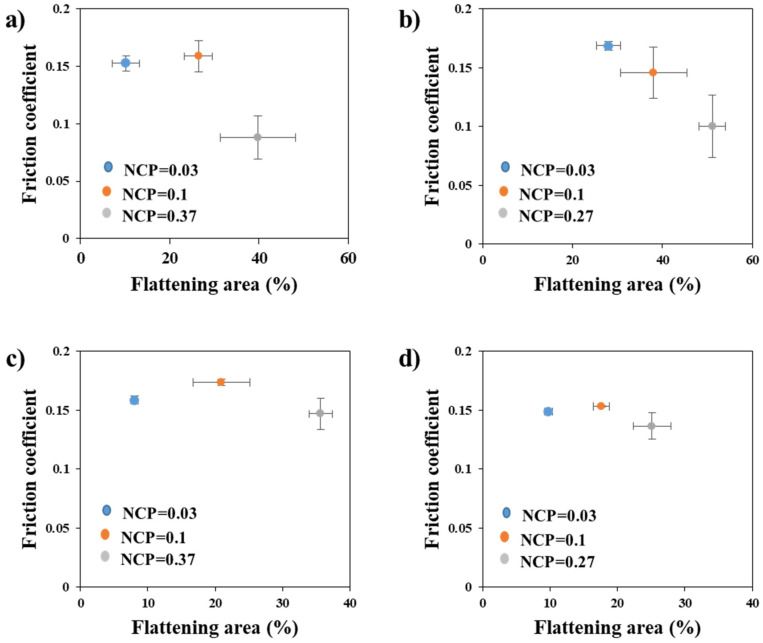
Friction coefficient vs. flattening area (%) under different normalized contact pressure: (**a**) TS340-GI, (**b**) TS980-GI, (**c**) TS340-GA, and (**d**) TS980-GA.

The experiments showed that the asperity flattening depended highly on the applied contact pressure. The detailed results on the relationship between the friction coefficient and contact condition, coating layer, and substrate material are summarized in Figure 12. For the low normalized contact pressure of 0.03, the friction coefficient linearly decreased as the flattening area increased. However, it increased at the normalized contact pressure of 0.1 in the case of TS340-GI. When the contact pressures were maximum (0.3 and 0.27 for 340 MPa and 980 MPa steel, respectively), the overall friction coefficients were sharply decreased, especially for the GI-coated specimens. However, there was no meaningful relationship between the friction coefficient and flattening area. The most noticeable change in friction coefficient could be observed in the TS340-GI steel when the normalized contact pressure was 0.3. In this case, the friction coefficient was reduced even under 0.06 from 0.14 at the normalized contact pressure of 0.1. For the GA-coated steels, the overall effect of the contact pressure on the friction coefficient was less. These results indicate that the abrupt reduction of the friction coefficient, which cannot be simply estimated from the empirical formula in terms of contact pressure, might be attainable in the sheet-forming process if the coating and process conditions are properly controlled.

## 5. Conclusions

The effect of contact characteristics such as the contact pressure and sliding velocity on the frictional behavior was investigated for two types of zinc coating on two strength-grade steel sheets. The two coating conditions, GI and GA coatings, were applied to 340 MPa and 980 MPa strength steels. To analyze the effect of the strength of substrate steel, the contact pressure normalized by their tensile strength was used. More in-depth analysis of the friction was provided in relation to the surface characteristics, such as fractography analysis, coating layers, and asperity flattening behavior. The main findings of the present study can be summarized as follows.

(1) As the normalized contact pressure increased, the area of asperity flattening increased consistently regardless of the sliding velocity, substrate material, and coating condition. This is clearer and consistent in the case of GI-coated steels. At a normalized contact pressure of 0.37, the flattening area reached approximately 50% for TS340-GI and 47% for TS980-GI, confirming that higher pressure promotes surface deformation. In contrast, GA-coated steels exhibited lower asperity flattening under similar conditions, likely due to the higher strength of the coating. Meanwhile, the effect of sliding velocity on the asperity flattening was rather minor without a clear tendency.
(2) However, for the GA-coated steels, the increase of the asperity flattening area was less than that of the GI-coated steels under a similar magnitude of normalized contact pressure. The difference in the effect of contact pressure on the asperity flattening can be related to the strength of the coating layer. The greater influence of contact pressure is attributed more to the softer GI-coating layer than the GA coating.
(3) The friction coefficient can be highly related to the material strength and coating layer combination. For example, an abrupt drop in friction coefficient could be observed when the asperity flattening area exceeded a certain critical limit. The most significant reduction occurred at the highest normalized contact pressure of 0.37, where the friction coefficient dropped by over 30%. In comparison, GA-coated steels exhibited a more stable friction coefficient with less variation across different contact pressures and sliding velocities. The friction coefficient for GA-coated steels ranged between 0.125 and 0.177, while GI-coated steels showed a wider range between 0.059 and 0.18.
(4) Therefore, in the presence of a lubrication system, it is plausible that a specific regime of contact condition may exhibit a considerable reduction of friction coefficient despite the enlarged occurrence of asperity flattening caused by sliding. This result indicates that the abruptly low friction coefficient, which may not be simply estimated from the conventionally utilized empirical formula based on the contact pressure, can exist in the industrial forming process.

## Figures and Tables

**Table 1:** Specimens used in the experiments.

Specimen	Tensile Strength (TS), MPa		Hardness, HV	Roughness, μm		
	Base Metals	Coatings	Coatings	Ra	Rz	Rsk
TS 340-GI	340	34 ± 5	102 ± 16	0.92	5.32	−0.39
TS 340-GA	340	89 ± 14	266 ± 43	1.16	8.34	−0.63
TS 980-GI	980	34 ± 5	102 ± 16	0.85	6.90	−0.74
TS 980-GA	980	89 ± 14	266 ± 43	1.18	8.65	0.00

**Table 2:** Contact pressure and its normalized value by the tensile strength of materials.

TS 340		TS 980	
Contact Pressure, MPa	Normalized Contact Pressure	Contact Pressure, MPa	Normalized Contact Pressure
10	0.03	33	0.03
33	0.10	100	0.10
130	0.37	267	0.27

## Data Availability

The original contributions presented in the study are included in the article, further inquiries can be directed to the corresponding author/s.

## Abbreviation

GI	galvanized
GA	galvannealed
SEM	scanning electron microscopy
EDS	energy dispersive spectroscopy
NCP	normalized contact pressure
EHL	elasto-hydrodynamic lubrication

## Appendix A

Detailed data for the characteristics of frictional behavior. The following figures and tables present the measured friction coefficients and frictional characteristics under various contact conditions.

**Table A1:** Frictional characteristics of tested specimens: (**a**) TS340-GI, (**b**) TS980-GI, (**c**) TS340-GA, and (**d**) TS980-GA.

Sliding Velocity	Normalized Contact Pressure	Friction Mode			Wear (Fracture)		
		Flattening (%)	Stick–Slip	Ploughing	Parallel Crack	Vertical Crack	Spalling
(a)							
0.05	0.03	6.69	medium		X	X	X
0.2	0.03	8.96	medium		X	X	X
0.9	0.03	14.97	small		X	X	X
0.05	0.1	31.52	large		X	X	X
0.2	0.1	24.57	large		X	X	X
0.9	0.1	23.28	large		X	X	X
0.05	0.37	50.20	small		X	X	X
0.2	0.37	42.41	medium		X	X	X
0.9	0.37	26.64	small		X	X	X
(b)							
0.05	0.03	24.23	medium		O	X	X
0.2	0.03	28.11	large		O	X	X
0.9	0.03	31.58	medium		O	X	X
0.05	0.1	26.12	small		O	X	X
0.2	0.1	45.86	small		O	X	X
0.9	0.1	41.98	small		O	X	X
0.05	0.27	46.72	small		O	X	X
0.2	0.27	55.08	small		O	X	X
0.9	0.27	51.39	small		O	X	X
(c)							
0.05	0.03	7.43	medium		△	△	X
0.2	0.03	8.15	medium		O	O	X
0.9	0.03	8.55	medium		O	O	O
0.05	0.1	27.65	small		O	△	O
0.2	0.1	18.82	small		O	O	O
0.9	0.1	16.24	small		O	O	O
0.05	0.37	35.69	small		△	O	O
0.2	0.37	38.06	small		O	O	O
0.9	0.37	33.14	small		O	O	O
(d)							
Sliding velocity	Normalized contact pressure	Friction mode			Wear (fracture)		
		Flattening (%)	Stick–slip		Parallel crack	Vertical crack	Spalling
0.05	0.03	4.64	small		X	O	X
0.2	0.03	9.37	small		△	O	X
0.9	0.03	13.7	small		O	O	X
0.05	0.1	17.7	small		△	O	X
0.2	0.1	19.85	small		O	O	O
0.9	0.1	15.75	small		O	O	O
0.05	0.27	20.74	small		O	O	O
0.2	0.27	26.07	small		O	O	O
0.9	0.27	28.49	small		O	O	O

Note: X: barely observed, O: almost always observed, △: occasionally observed.
